# Real-space navigation in patients with focal epilepsy underpins the prominent role of the hippocampus for allocentric navigation in humans

**DOI:** 10.1007/s00415-026-14164-7

**Published:** 2026-09-27

**Authors:** Denis Grabova, Elisabeth Kaufmann, Cauchy Pradhan, Jan Rémi, Christian Vollmar, Ilias Masouris, Elisabeth Wlasich, Julia Kustermann, Andreas Zwergal, Florian Schöberl

**Affiliations:** 1 Department of Neurology, LMU University Hospital, LMU Medizin, LMU Munich, Munich, Germany; 2 German Center for Vertigo and Balance Disorders (DSGZ), LMU University Hospital, LMU Medizin, LMU Munich, Munich, Germany

**Keywords:** Navigation, Spatial orientation, Hippocampus, Epilepsy, Cognitive domain

## Abstract

**Background:**

An in-depth assessment of spatial navigation in patient cohorts with focal brain disorders may inform about the functional topography of underlying brain networks. The aim of the current study was to investigate the role of the right and left hippocampi on recombining novel routes in a real-space environment as a correlate for allocentric navigation abilities in patients with focal epilepsies.

**Methods:**

In a single-center approach, adults with unifocal epilepsy were prospectively recruited from the tertiary epilepsy center at LMU Munich. 16 patients with right-sided mesial temporal lobe epilepsy (rTLE), 16 with left-sided mesial temporal lobe epilepsy (lTLE), 15 with frontal lobe epilepsy (FLE), and 24 age-matched healthy controls conducted a real-space navigation paradigm that required recognition of familiar routes and recombination of novel routes. Visual exploration behavior and navigation trajectories were documented by a gaze-controlled head-fixed camera and analyzed post hoc across groups and navigation conditions.

**Results:**

Epilepsy patients (mean age 40.7 years) performed significantly worse in finding navigation targets than controls (*p*<0.001). Age, duration of epilepsy, and structural brain lesions affected navigation performance. After correcting for confounders, rTLE patients showed significantly inferior performance compared to FLE, especially for recombined novel routes. lTLE patients were impaired mainly on recombined novel routes. rTLE patients spent more time at crossroads and used shortcuts less often than controls.

**Conclusion:**

This study supports the hippocampus as a major hub for human goal-oriented navigation in real environments, with right hippocampal dysfunction particularly impairing allocentric navigation and creation of novel routes.

**Supplementary Information:**

The online version contains supplementary material available at https://doi.org/10.1007/s00415-026-14164-7.

## Introduction

Target-oriented navigation is a distinct higher-order brain function requiring a complex cerebral network to continuously update and integrate idio- and allothetic multisensory information for egocentric or allocentric navigation strategies [1–5]. Egocentric navigation means compass-like representation of external objects and locations in the environment in direct relation to the navigator’s current position. In contrast, allocentric navigation enables relating spatial information of objects to each other independent of the current position in space by a map-like mental representation [10, 11]. There is converging evidence that hippocampus and entorhinal cortex are mandatory to create and retrieve such a cognitive map of the environment, which enables creating novel routes, flexible wayfinding, and optimizing routes by applying shortcuts [3, 11, 12]. Furthermore, spatial navigation in humans seems to be primarily a cognitive domain of the non-language dominant, i.e., right hemisphere [6–9].

In adults with well-characterized focal epilepsies, the impact of restricted lesions and/or dysfunction within the cerebral navigation network on navigation performance and strategy in real space can be optimally examined. In several recent studies, deficits in allocentric navigation were found in patients with mesial temporal lobe epilepsy [13–16]. However, it is still under debate, whether spatial navigation abilities are more severely affected in right-sided than left-sided mesial temporal lobe epilepsy [17, 18].

To assess the role of the right and left hippocampi on recombining novel routes in a real-space environment as a correlate for allocentric navigation, we examined three different patient cohorts, i.e., right-sided mesial temporal lobe epilepsy (rTLE), left-sided mesial temporal lobe epilepsy (lTLE), and frontal lobe epilepsy (FLE), by means of a well-established real-space navigation paradigm. By simultaneous tracking of eye and head movements with a head-mounted eye tracking system, we were able to analyze error rates in familiar and recombined novel routes separately as well as visual exploration behavior and walked trajectories [19–22]. We hypothesized that patients with rTLE will have most severe deficits in allocentric navigation performance.

## Materials and methods

### Subjects

The study cohorts were recruited from the tertiary epilepsy center at LMU University Hospital. All patients underwent a detailed multimodal electrophysiological and imaging assessment to localize their epileptogenic zone. Only patients with definite unifocal epilepsy and a low-seizure frequency on antiepileptic medication (i.e., ≤ one focal or generalized seizure per month) were included.

### Neuropsychological testing

All participants performed different subtests of the Consortium to Establish a Registry for Alzheimer’s Disease (CERAD) plus test battery for the evaluation of verbal memory and executive function, including word-list learning (delayed recall), Trail-Making Test Part B (TMT-B), and semantic word fluency [23]. Furthermore, the Rey–Osterrieth complex figure test (ROCF) for the evaluation of visuospatial constructional abilities and visual memory and the 4 Mountains Test (4MT), a short computerized mental rotation task, were performed in each participant [24, 25].

### Real-space navigation paradigm

**Fig. 1:**
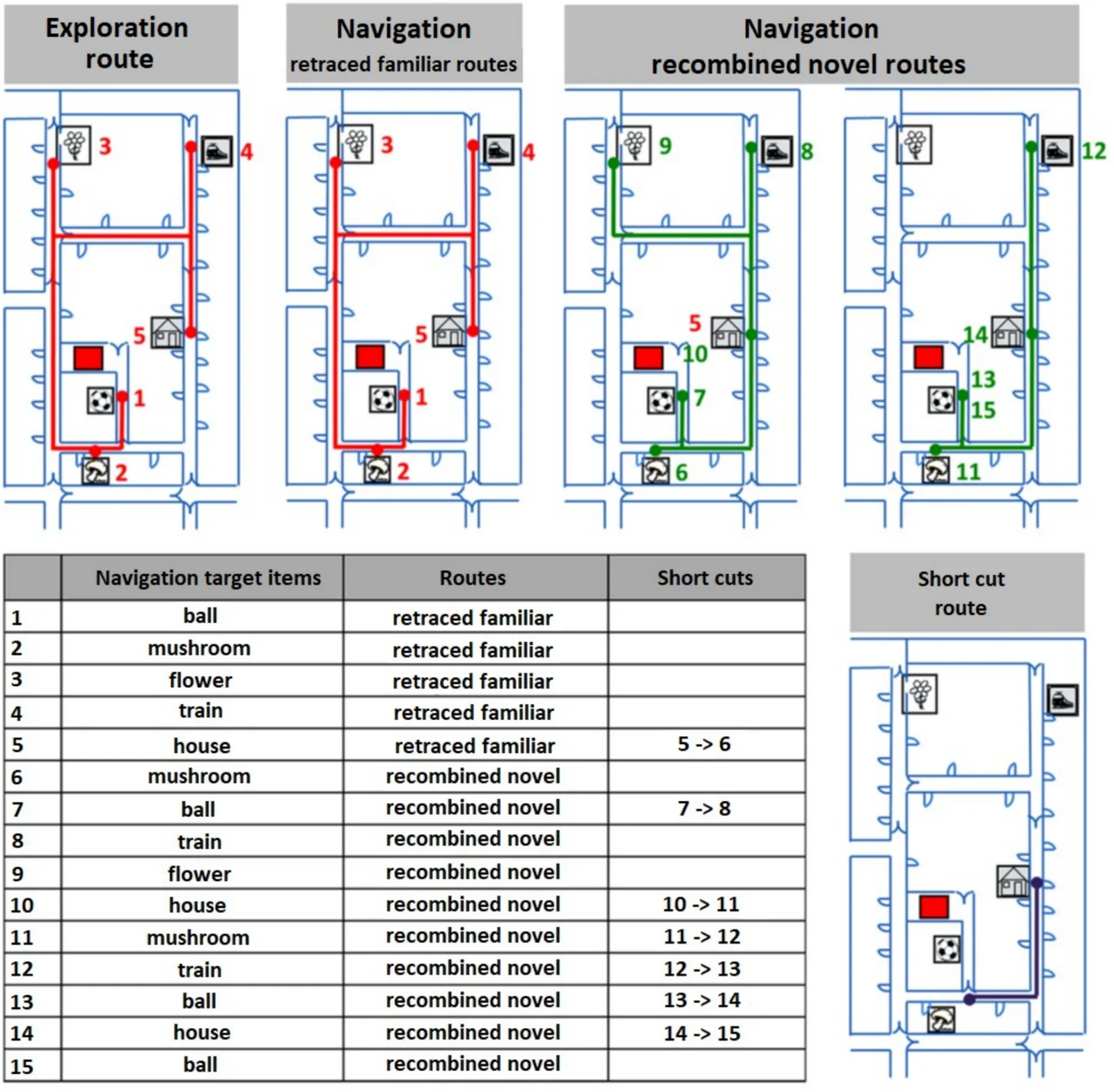
Navigation paradigm in real space. All participants performed a real-space navigation paradigm in a complex and unfamiliar spatial environment to test their spatial orientation abilities. The area, in which five target items (pictures of ball/mushroom/flower/train/house) had been placed, was shown to the participants during an examiner-guided walk in the first part of the paradigm (exploration, upper panel, left side). In the second part, participants were instructed to find the items in a predefined pseudo-randomized order over 10 min beginning from the same starting spot (red square). The first five routes in the navigation paradigm were the same as the previously explored routes and thus had to be simply retraced (upper panel, middle). By changing the order of the next 10 target items, the recombination of novel routes was required (upper panel, right side). Possible shortcut routes were registered (lower panel, right side). The order of items during navigation is written in a table (lower panel, left side) and appears as numbers next to the target items in the figures (adapted from reference [19, 21])

All participants performed a well-established navigation paradigm in a complex and unfamiliar environment to test their spatial navigation performance (Fig. 1) [19, 20]. In the examiner-guided exploration phase, five target items placed in the 700m^2^ area of an outpatient clinic were shown to the participant in a defined order along defined routes. In the directly following navigation phase, participants had to navigate toward 15 target items in 10 minutes in a pseudo-randomized sequence determined by the examiner. As the first five routes toward the target items were identical to the exploration phase, they were defined as familiar retraced routes. The pseudo-randomized order of the next 10 target items was designed to involve the planning of novel routes and the potential use of shortcuts through previously unexplored territory (i.e., recombined novel routes), thus requiring an allocentric strategy. In case of an excellent test performance with the successful completion of the navigation paradigm below 10 minutes, further target items, which required planning of recombined novel routes, had to be found until completion of altogether 10 minutes of the navigation phase. By recalling the 5 target items after exploration and navigation, an impairment in navigation performance due to (verbal) memory dysfunction was excluded. The primary outcome parameter was the error rate in all subgroups. Error rates were normalized to 15 routes in total in our navigation task (normalized error rate = (15 – correctly found items)/15 × 100%). Error rates were further differentiated for retraced familiar (egocentric) and recombined novel (allocentric) routes and normalized to the expected number of target items in the task (retraced familiar routes: *n* = 5, recombined novel routes: *n* = 10). The total use of shortcuts divided by the number of all possible shortcuts defined the percentage use of shortcut routes. Traveled distances were set in relation to the optimized path length to quantify inefficient route finding (Fig. 1).

### Recording of navigational path and visual exploration behavior

Participants wore a gaze-monitoring head camera during the experiment to document their visual exploration behavior [26]. Post hoc analysis of the recorded videos was used to determine the error rates for finding target items on retraced familiar (egocentric) and recombined novel (allocentric) routes of the navigation paradigm as well as the total error rates. The search path during the navigation task was mapped by accumulating time at a specific place and was analyzed quantitatively for use of shortcuts, and time spent at crossroads. The parameter traveled distance/optimal path (%) was used as an indicator for inefficient route finding.

Saccade and fixation analysis was performed with MATLAB2012a (Mathworks, Natick, MA) software applying an established algorithm [27]. Raw eye movements in the x- and y-axes were converted into degrees and displayed as heat maps. The overall distance traversed by gaze was determined as follows:

$$\sqrt {\left( {{{xt1} \mathord{\left/ {\vphantom {{xt1} {xt2}}} \right. \kern-0pt} {xt2}}} \right)^{2} + \left( {yt1 - yt2} \right)^{2} }$$

where t1 and t2 refer to adjacent time points. Velocity was determined as distance/time, acceleration as (velocity_t1_ − velocity_t2_)/time. A velocity of >240°/s and an acceleration >3000°/s^2^ defined a saccade. Fixations were events of >100-millisecond duration at an eye velocity of <240°/s and acceleration <3000°/s^2 19^. The number and frequency of saccades and fixations (Hz) were computed quantitatively for the whole paradigm and for retraced familiar routes and recombined novel routes respectively [19, 26, 27]. Post hoc annotation of saccades and fixations to objects was performed manually by an experienced investigator. All the fixation targets were categorized: (1) unspecific saccades and fixations (e.g., floor, wall, ceiling), (2) task-inherent saccades and fixations (e.g., investigator, instructions, target items), saccades and fixations reflecting general search behavior (e.g., doors, views into rooms/corners/niches), and (3) saccades and fixations toward objects suitable as landmarks for (re)orientation (e.g., distinct fixed pictures, objects and furniture), which were defined as object saccades and fixations. The number and frequency of object saccades and fixations (Hz) were evaluated for the whole navigation paradigm as well as for retraced familiar and recombined novel routes separately [22].

We performed quantitative analyses for the following parameters: number of fixations and saccades during the total real-space navigation paradigm, retraced familiar routes, and recombined novel routes, separately. To correct for differences in locomotion speed between participants, fixations and saccades were expressed in Hz (absolute values/s).

### Statistical analysis

Behavioral and navigational measurements of the four study subgroups (rTLE, lTLE, FLE, and HC) were analyzed using JASP (0.16.3; JASP Team, 2022) [28]. One-way analysis of variance (ANOVA) was used to compare demographic data, neuropsychological test performance, navigation performance, visual exploration behavior, and navigation strategy. Šidák post hoc test was applied to analyze differences between groups. Correlation analyses for the effects of age, duration of focal epilepsy, and underlying etiology of the focal epilepsy (i.e., structural brain lesion/no structural brain lesion) on navigation performance (error rates) were conducted using Spearman’s rho. Analysis of covariance (ANCOVA) was applied to compare navigation performance (error rates) between the epilepsy subgroups corrected for possible confounders (i.e., age, lesion, duration of epilepsy). To correct for multiple testing, Šidák correction was applied as a post hoc test for ANOVA and ANCOVA. *P*-values <0.05 were considered statistically significant for all the described statistical analyses.

## Results

### Patient characteristics

The study cohort was composed of the following subgroups: 16 patients with rTLE (mean age: 50.1±15.0 years), thereof three with non-lesional epilepsy and 13 with underlying structural lesions (i.e., hippocampal sclerosis, cortical dysplasia etc.); 16 patients with lTLE (mean age: 37.5±12.1 years), thereof ten with structural lesions; 15 patients with FLE (mean age: 33.7±9.0 years), thereof eight with structural lesions (Table 1). 6/16 patients with rTLE, 5/16 with lTLE, and 3/15 patients with FLE had undergone previous epilepsy surgery. Procedures in the TLE groups comprised amygdalohippocampectomy and anterior temporal resections, whereas focal resections of the identified epileptic zone(s) were performed in the FLE patients (Table 1). The control cohort consisted of 24 healthy controls (HC) (mean age: 35.4±10.3 years) without a neurologic or psychiatric medical history, with a normal neurologic status and unremarkable brain structure on magnetic resonance imaging (MRI). rTLE patients were slightly older than lTLE patients (*t*=3.1, *p*=0.021), FLE patients (*t*=3.8, *p*=0.002), and HC (*t*=3.9, *p*=0.002). There was no difference in the distribution of handedness between the groups.

**Table 1:** Patient characteristics

Patient	Age	Sex	Diagnosis	Etiology	Age at onset	Epilepsy disease duration	Number of antiepileptic drugs	Epilepsy surgery	Seizure frequency after surgery
1	63	M	TLE right	Hippocampus sclerosis right	11	52 years	1	Amygdalohippo-camptectomy	Reduction of seizures
2	73	F	TLE right	Hippocampus sclerosis right	53	20 years	2		
3	20	F	TLE right	Hippocampus sclerosis right	9	11 years	1	Anterior partial temporal lobe resection	Reduction of seizures
4	48	M	TLE right	Cortical dysplasia right mesiotemporal	4	44 years	3		
5	39	M	TLE right	Hippocampus sclerosis right	38	1 year	1	Anterior partial temporal lobe resection	Seizure-free
6	33	F	TLE right	Hippocampus sclerosis right	15	18 years	2		
7	44	F	TLE right	Hippocampus sclerosis right	27	17 years	1		
8	38	F	TLE right	Hippocampus sclerosis right	24	14 years	2	Temporal pole resection	Reduction of seizures
9	60	F	TLE right	Hippocampus sclerosis right	30	30 years	1		
10	61	M	TLE right	No lesion	58	3 years	1		
11	37	M	TLE right	No lesion	37	3 months	1	Microsurgical amygdala resection	Seizure-free
12	39	F	TLE right	Hippocampus sclerosis right	10	29 years	1		
13	64	F	TLE right	Hippocampus sclerosis right	10	54 years	3		
14	55	F	TLE right	No lesion					
15	70	M	TLE right	Hippocampus sclerosis right	50	20 years	1		
16	57	M	TLE right	Hippocampus sclerosis right	55	2 years	2	Anterior pole resection with amygdalohippo-camptectomy	Seizure-free
17	37	F	TLE left	Hippocampus sclerosis left	9	28 years	4	Anterior pole resection withAmygdalohippo-camptectomy	Seizure-free
18	34	M	TLE left	Hippocampus sclerosis left	6	28 years	1	Amygdalohippo-amptectomy	Seizure-free
19	41	M	TLE left	Hippocampus sclerosis left	34	7 years	2		
20	25	F	TLE left	Hippocampus sclerosis left	15	10 years	1		
21	27	F	TLE left	After glioma resection left temporal	20	7 years	2		
22	32	M	TLE left	Cortical dysplasia left mesiotemporal	1	31 years	2	Amygdalohippocampectomy	Seizure-free
23	31	F	TLE left	No lesion	6	25 years	2		
24	27	F	TLE left	No lesion	25	2 years	2		
25	20	F	TLE left	Posthemorrhagic defect left temporal	18	2 years	1		
26	63	M	TLE left	Hippocampus sclerosis left	25	38 years	2	Anterior pole resection with amygdalohippocampectomy	Seizure-free
27	38	F	TLE left	No lesion	37	1 year			
28	29	F	TLE left	No lesion	29	3 months	1		
29	50	F	TLE left	Hippocampus sclerosis left	48	2 years	1	Temporal lobe resection with amygdalohippocampectomy	Reduction of seizures
30	54	F	TLE left	No lesion					
31	54	F	TLE left	No lesion	53	1 year	2		
32	38	M	TLE left	Cortical dysplasia left temporal	36	2 years	1		
33	44	M	FLE right	Cortical dysplasia right frontal	38	6 years	1	Focal resection	Seizure-free
34	29	M	FLE right	Cortical dysplasia right frontal	15	14 years	2	Focal resection	Seizure-free
35	35	M	FLE right	Cortical dysplasia right frontal	10	25 years	5		
36	26	M	FLE right	No lesion	19	7 years	1		
37	41	F	FLE right	No lesion	34	7 years	1		
38	28	M	FLE left	Cortical dysplasia left frontal	6	22 years	2	Focal resection	Seizure-free
39	53	M	FLE left	Posttraumatic gliosis left frontal	18	35 years	2		
40		M	FLE left	No lesion					
41	37	M	FLE left	Cortical dysplasia left frontal	6	31 years	3		
42	39	M	FLE left	No lesion	7	32 years	2		
43	38	M	FLE left	No lesion	10	28 years	3		
44	19	F	FLE left	No lesion	15	14 years			
45	31	F	FLE left	No lesion	7	24 years	1		
46	26	M	FLE bilateral	Cortical dysplasia right frontal	17	9 years	3		
47	26	M	FLE bilateral	No lesion	2	24 years	4		

### Psychometric measures

**Fig. 2:**
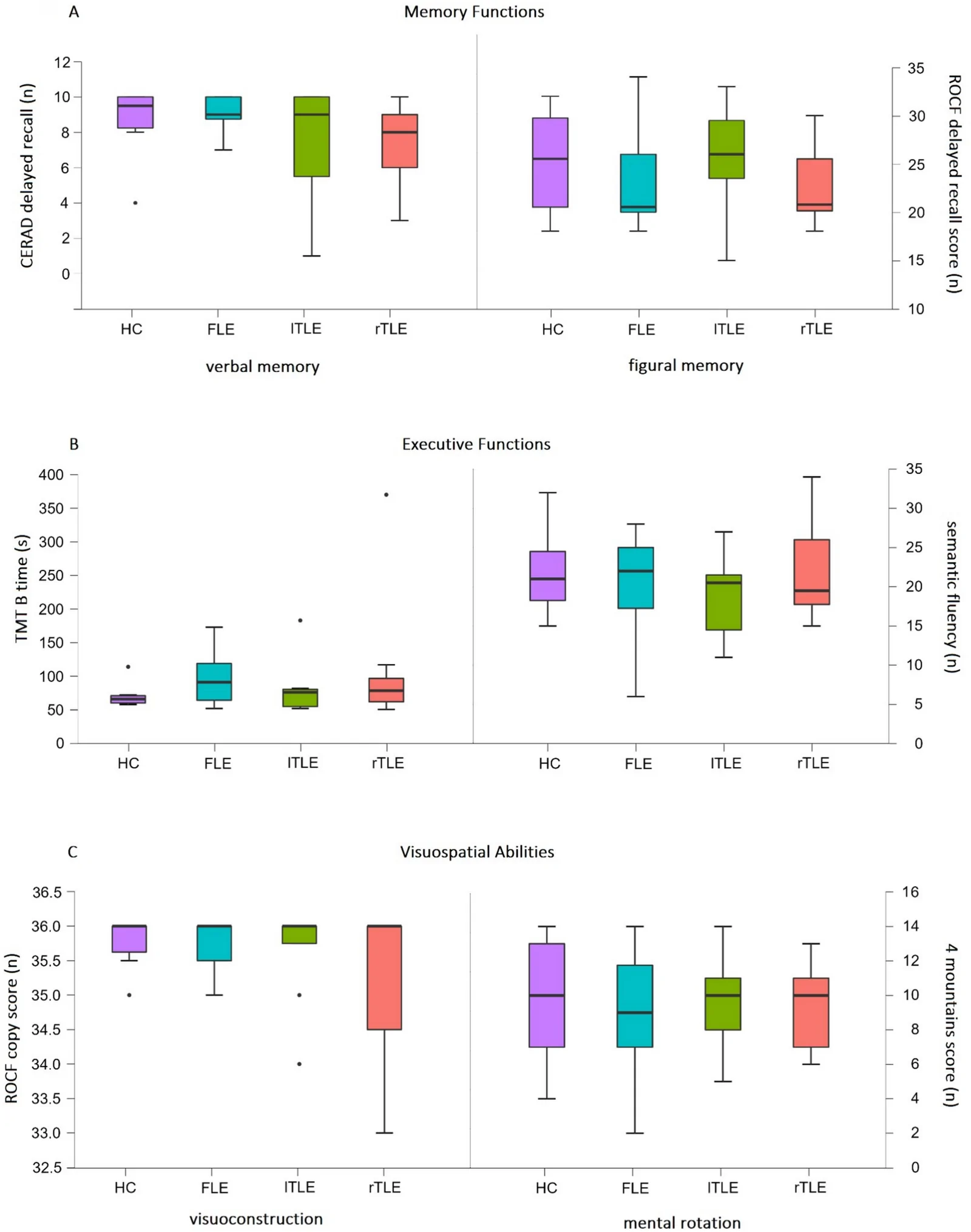
Psychometric measures in the different patient groups with epilepsy and healthy controls. **A** Memory function: Performances for delayed recall of the Rey–Osterrieth Complex Figure Test (ROFC) and delayed recall of the Consortium to Establish a Registry for Alzheimer’s Disease (CERAD) word list did not differ between subgroups. **B** Executive function: Performances for Trail-Making Test Part B (TMT-B) and semantic verbal fluency did not differ between subgroups. **C** Visuospatial abilities: Performances for the copy of the ROFC and for the 4 Mountains Test were also similar between subgroups. *rTLE* right-sided mesial temporal lobe epilepsy, *lTLE* left-sided mesial temporal lobe epilepsy; *FLE* frontal lobe epilepsy; *HC* healthy controls

Performances for delayed recall of the word list, TMT-B, semantic verbal fluency, and delayed recall of the ROCF did not differ between the different patient groups with rTLE, lTLE, FLE, and HC (Fig. 2, for details, see Suppl. Table 2). Performances in the 4MT were also similar between the different patient groups and HC; however, patients with FLE needed significantly more time to absolve the task than HC (*t*=3.0, *p*=0.030).

### Navigation performance of epilepsy patients and healthy controls

The total normalized error rate during real-space navigation was significantly higher in the rTLE patient group (71.7±16.8%) compared to lTLE patients (45.3±30.8%) (*t*=3.3, *p*=0.013), FLE patients (28.6±16.2%) (*t*=4.8, *p*<0.001), and healthy controls (−3,6±8.3%) (*t*=10.3, *p*<0.001). Furthermore, lTLE (*t*=6.4, *p*<0.001) and FLE patients (*t*=3.7, *p*=0.004) performed significantly worse than HC.

**Fig. 3:**
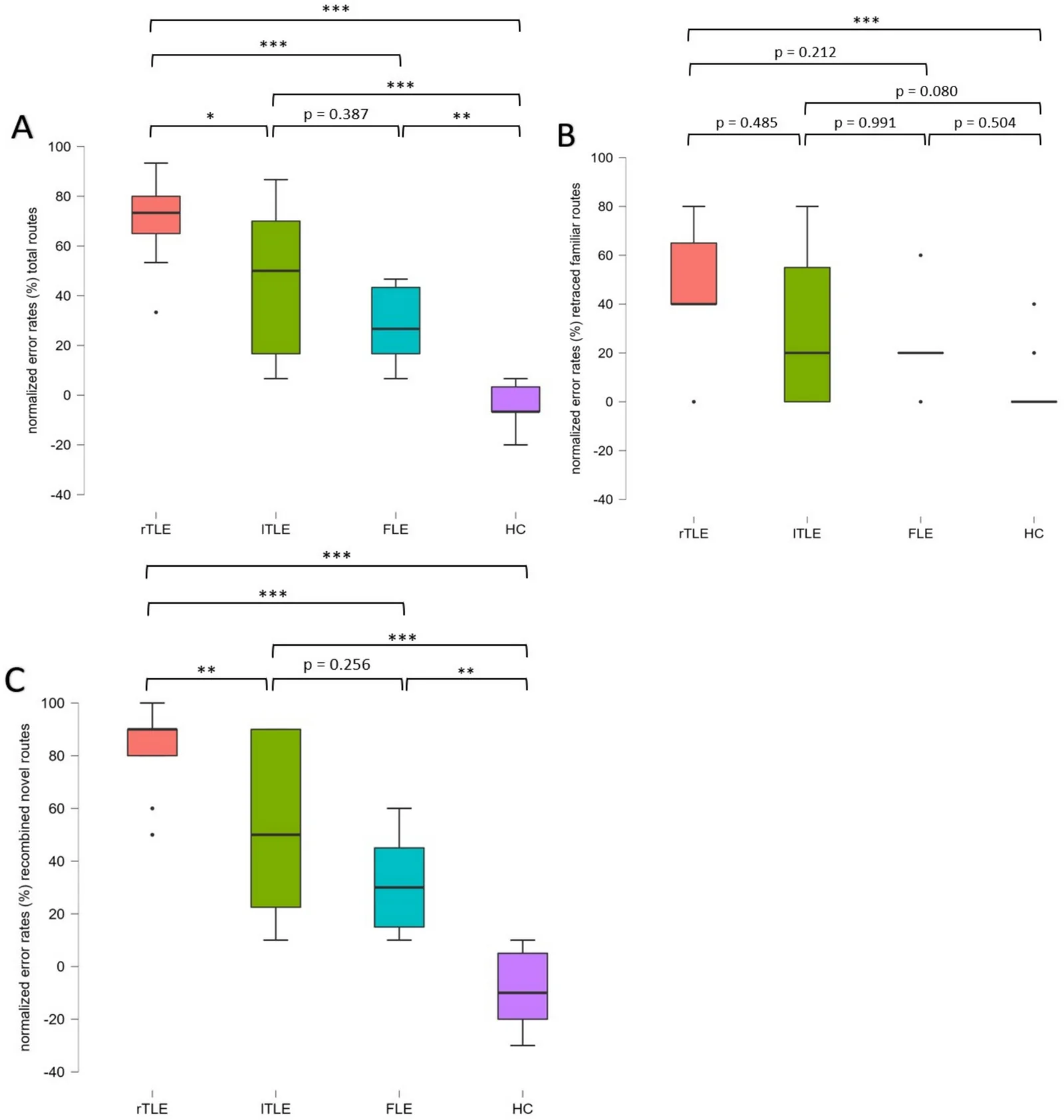
Navigation performance in the different epilepsy groups and healthy controls. The total normalized error rate during the navigation paradigm was significantly higher in rTLE patients compared to lTLE patients (*p*=0.013), FLE patients (*p*<0.001) and HC (*p*<0.001). rTLEpatients made more errors when recombining novel routes than lTLE patients (*p*=0.009), FLE patients (*p*<0.001) and healthy controls (*p*<0.001), while their performance did not differ on retracing familiar routes compared to lTLE (*p*=0.485) and FLE patients (*p*=0.212). Normalized error rates are depicted as Tukey plots in %. *p<0.05, **p<0.01, ***p<0.001. *rTLE* right-sided mesial temporal lobe epilepsy, *lTLE* left-sided mesial temporal lobe epilepsy, *FLE* frontal lobe epilepsy, *HC* healthy controls

Navigation performance on retraced familiar routes did not differ significantly between rTLE patients (46.7±27.4%) and lTLE (30.0±33.0%) (*t*=1.7, *p*=0.485) or FLE patients (22.9±18.0%) (*t*=2.1, *p*=0.212), respectively. However, on recombined novel routes, rTLE patients (84.2±15.1%) performed significantly worse than lTLE (53.0±34.7%) (*t*=3.4, *p*=0.009) and FLE patients (31.4±21.2%) (*t*=5.2, *p*<0.001). Both, on retraced familiar routes and on recombined novel routes, error rates between rTLE patients and HC differed significantly (retraced: *t*=9.1, *p*<0.001, recombined: *t*=11.1, *p*<0.001). FLE patients (*t*=4.0 *p*=0.002) and lTLE patients (*t*=7.0, *p*<0.001) also performed significantly worse than HC on recombined novel routes contrasting with comparable performances on retraced familiar routes (Fig. 3, Table 2).

**Table 2.** Navigational and visual exploration parameters in rTLE, lTLE, and FLE patients and HC

Parameter	rTLE	lTLE	FLE	HC	ANOVA (F, p)
Total paradigm: error rates (%)	71.7±16.8	45.3±30.8	28.2±16.2	−3.6±8.3	37.3, <0.001*
Familiar routes: error rates (%)	46.7±27.4	30.0±33.0	22.9±18.0	5.3±11.9	7.1, <0.001*
Novel routes: error rates (%)	84.2±15.1	53.0±34.7	31.4±21.2	−8.0±13.2	43.3, <0.001*
Total paradigm: items found (*n*)	4.7±2.9	9.3±4.4	12.0±3.4	14.4±2.4	29.4, <0.001*
Familiar routes: items found (*n*)	2.7±1.3	3.7±1.4	4.2±0.9	4.4±0.9	7.3, <0.001*
Novel routes: items found (*n*)	1.9±1.9	5.7±3.4	7.8±2.8	10.0±1.8	35.0, <0.001*
Time at unexplored stretch (%)	13.3±6.3	5.1±6.3	6.3±6.9	9.2±7.2	3.1, 0.037*
Duration at crossroads (%)	28.1±5.6	20.9±7.8	21.9±6.9	19.7±2.5	5,5, 0.003*
Shortcuts used (%)	13.9±23.1	20.7±36.2	27.8±31.0	65.0±44.1	5.5, 0.003*
Traveled distance/optimal path (%)	138.2±48.0	128.9±31.6	131.8±27.7	98.3±105.0	2.5, 0.080
Total paradigm: saccades/time (Hz)	4.3±1.4	4.4±1.2	3.9±1.2	3.0±1.3	3.4, 0.028*
Total paradigm: fixations/time (Hz)	2.2±0.3	2.3±0.5	2.5±0.2	2.3±0.2	1.0, 0.425
Familiar routes: saccades/time (Hz)	4.1±1.4	4.1±1.1	3.5±1.1	2.7±1.4	3.2, 0.035*
Familiar routes: fixations/time (Hz)	2.2±0.2	2.3±0.5	2.3±0.2	2.2±0.6	0.3, 0.859
Novel routes: saccades/time (Hz)	4.1±1.4	4.0±1.4	4.0±1.0	2.9±1.3	2.5, 0.072
Novel routes: fixations/time (Hz)	2.2±0.2	2.2±0.6	2.3±0.2	2.3±0.3	0.6, 0.630
Total paradigm: object saccades/time (Hz)	0.9±0,7	0.8±0.5	0.9±0.8	1.2±0.4	1.1, 0.350
Total paradigm: object fixations/time (Hz)	0.5±0.2	0.6±0.3	0.7±0.4	1.2±0.3	9.1, <0.001*
Familiar routes: object saccades/time (Hz)	1.0±0.7	0.9±0.5	0.8±0.6	1.2±0.4	1.3, 0.287
Familiar routes: object fixations/time (Hz)	0.5±0.3	0.6±0.3	0.6±0.4	1.2±0.3	11.3, <0.001*
Novel routes: object saccades/time (Hz)	0.7±0.5	0.9±0.4	1.0±0.9	1.3±0.6	1.7, 0.191
Novel routes: object fixations/time (Hz)	0.5±0.2	0.7±0.3	0.8±0.5	1.3±0.5	8.5, <0.001*

*Significant inter-group differences in one-way analysis of variance (ANOVA) testing. Post hoc Šidák test was applied for the correction of multiple comparisons

*rTLE* right-sided mesial temporal lobe epilepsy, *lTLE* left-sided mesial temporal lobe epilepsy, *FLE* frontal lobe epilepsy, *HC* healthy controls, *ANOVA* analysis of variance, *Hz* Hertz

The total normalized error rates increased significantly with higher age (Rho=0.365, *p*=0.015), a longer duration of focal epilepsy (Rho=0.399, *p*=0.032) and the existence of a structural brain lesion as underlying cause of the focal epilepsy (Rho=0.587, *p*<0.001). By applying an ANCOVA to correct for all these confounders in the three epilepsy subgroups, there was a significantly higher total error rate and error rate for recombined novel routes in the rTLE group as compared to FLE patients (total routes: *t*=4.1, *p*=0.001; recombined routes: *t*=4.4, *p*<0.001). Additionally, lTLE patients exhibited a significantly inferior performance than FLE patients regarding recombined novel routes, exclusively (*t*=2.8, *p*=0.030). However, there was neither a significant difference in navigation performance for the total task nor in recombined novel routes any more between the rTLE and lTLE group when correcting for the described confounders (total routes: *t*=1.8; *p*=0.226; recombined routes: *t*=1.8; *p*=0.227).

### Visual exploration behavior and navigation strategy in epilepsy patients and healthy controls

Overall, saccades, fixations, and object saccades (i.e., saccades to objects feasible as landmarks for orientation) during the total paradigm, retraced familiar routes, and recombined novel routes were comparable between all subgroups in post hoc analysis (Table 2). rTLE, lTLE, and FLE patients exhibited less object fixations (i.e., fixations to objects feasible as landmarks for orientation) than HC in the total paradigm (rTLE: 0.5±0.2, *t*=−4.8, *p*<0.001) (lTLE: 0.6±0.3, *t*=−3.6, *p*=0.006) (FLE: 0.7±0.5, *t*=−2.9, *p*=0.037) (HC: 1.2±0.3), and on retraced familiar routes (rTLE: 0.5±0.3, *t*=−5.1, *p*<0.001) (lTLE: 0.6±0.3, *t*=−4.2, *p*=0.001) (FLE: 0.6±0.4, *t*=−3.7, *p*=0.004) (HC: 1.2±0.3). Moreover, on recombined novel routes, rTLE (0.5±0.2, *t*=−4.8, *p*<0.001) and lTLE patients (0.7±0.3, *t*=−3.2, *p*=0.018) used less object fixations than HC (1.3±0.5) with similar results between HC and FLE patients (0.8±0.5, *t*=−2.5, *p*=0.110) (Table 2).

In addition to the visual fixation behavior, the search path of rTLE patients differed significantly from other subgroups. rTLE patients (28.1±5.6%) spent more time at crossroads than lTLE patients (21.0±7.8%) (*t*=3.0, *p*=0.030) and HC (19.7±2.4%) (*t*=3.9, *p*=0.002) with similar results compared to FLE patients (21.9±6.9%) (*t*=2.3, *p*=0.151) (Table 2). Furthermore, rTLE patients (13.3±6.3%) spent more time in unexplored territory than lTLE patients (5.1±6.3%) (*t*=2.8, *p*=0.042), while spending similar time in unexplored territory as FLE patients (6.3±6.9%) (*t*=2.2, *p*=0.192) and HC (9.2±7.2%) (*t*=1.6, *p*=0.538). The targeted use of shortcuts was lower in rTLE (13.9±23.1%) (t=−3.7, *p*=0.004) and lTLE patients (20.7±36.2%) (*t*=−3.0, *p*=0.025) compared to HC (65.0±40.1%), while FLE patients (27.8±31.0%) (*t*=−2.2, *p*=0.203) used shortcuts similarly as HC. The traveled distance in relation to the optimal path did not differ significantly between the subgroups.

## Discussion

**Fig. 4:**
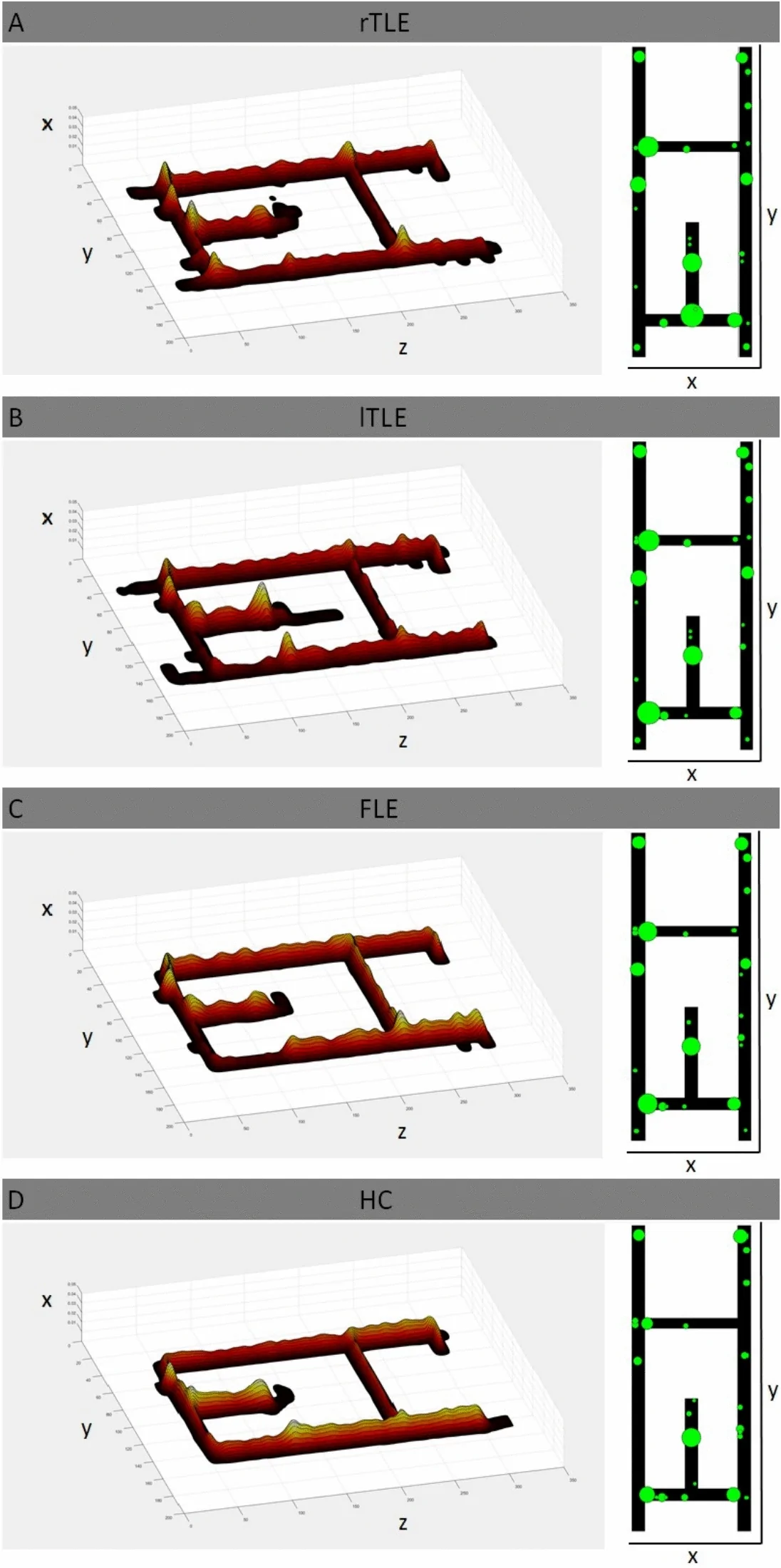
Navigation strategy and visual exploration behavior in rTLE-, lTLE-, FLE patients and HC. rTLE patients spent significantly more time at crossroads than TLE (*p*=0.030) and HC (*p*=0.002) and made less use of shortcuts than HC (*p*=0.004), indicating a deficit to create and retrieve a mental representation of the environment. The position of the 25 most frequently fixated objects feasible as landmarks in each group was indicated on a ground map as green circles (diameters representing the average number of views to a respective object across the whole group). *rTLE* right-sided mesial temporal lobe epilepsy; *lTLE* left-sided mesial temporal lobe epilepsy; *FLE* frontal lobe epilepsy; *HC* healthy controls

The major findings of this clinical cohort study were as follows: (1) TLE patients (rTLE and lTLE) performed significantly worse on the applied real-space navigation paradigm. (2) Increasing age, longer duration of focal epilepsy, and underlying brain lesions as the underlying cause of the epilepsy correlated with worse performance in all epilepsy subgroups. (3) TLE patients exhibited particular deficits on creating so far unknown routes. rTLE patients spent significantly more time at crossroads and unexplored territory and applied a possible shortcut within the real-space environment less often, even when directly compared with lTLE patients. This study underpins previous evidence that the hippocampus is a major hub within the human brain network for goal-oriented navigation in real environments and also confirms that regions within the frontal lobe play an important role within the human brain network for spatial navigation. Right hippocampal dysfunction seems to particularly impair the creation and execution of novel routes, which would emphasize its prominent role for allocentric navigation (Fig. 4).

In the current study, epilepsy per se seems to affect accuracy in a real-space navigation task. In line with previous studies, increasing age was a major factor for a worse performance in the applied real-space navigation task. As expected, a longer duration of epilepsy and the presence of structural epileptogenic brain lesions contributed to an inferior performance in the applied real-space navigation task in the whole group of epilepsy patients. Particularly, the recombination of novel, so far unknown routes, was significantly impaired in patients with epilepsy as compared to HC. Performance in the applied real-space navigation task was particularly inferior in patients with TLE, thus supporting the prominent role of the hippocampus for human navigation [1–4]. Consequently, TLE patients also performed significantly worse than FLE patients in the task. The particular function of the hippocampus seems to be the creation of a detail-rich mental representation of the surrounding environment, so called building up a “cognitive map” [2, 3, 12]. The observation that rTLE patients stayed longer at crossroads and also unexplored territory could indicate a comparably inaccurate representation of the environment in terms of either metric aspects (i.e., distances and directions) or in terms of the efficient selection and usage of distinct visual objects as landmarks for (re)orientation [2, 7, 8]. The additional finding that the possible shortcut within the applied real-space navigation task was obviously not realized in TLE patients would rather speak for an overall deficit to build up a cognitive map of the environment. Further support of this hypothesis derives from the eye tracking data with less object fixations (i.e., saccades toward distinct visual cues) in FLE, rTLE, and lTLE patients during the total paradigm and familiar routes, but less such fixations during the recombined novel routes only in the subgroup with TLE (i.e. rTLE and lTLE). In contrast, overall saccades did not differ significantly between subgroups. These results underline the view that human spatial navigation represents a complex and multifaceted cognitive function comprising different cognitive processes simultaneously [9–11]. This is in line with the multiple imaging data over the last two decades establishing the multiregional brain network for navigation in humans [2, 3]. Therefore, it does not seem surprising that frontal lobe dysfunction in FLE impairs spatial navigation abilities together with altered navigational behavior during our real-space task. Compared to HC, FLE patients exhibited deficits in recombining novel routes. Several explanations may be possible: distinct regions in the frontal lobe, particularly the dorsolateral prefrontal cortex (DRPFC), play a major role for goal-oriented planning and flexible wayfinding with correspondingly increased neuronal activation pattern during active navigation [1, 29–31]. In turn, a deficit on planning novel routes seems a plausible explanation of navigation deficits in FLE. Furthermore, frontal lobe regions also have extensive cortical interactions with the hippocampus. Thus, prefrontal brain activation patterns also might correlate with path integration accuracy, possibly contributing to an erroneous cognitive map formation [3, 32, 33].

In previous studies, lateralization of hippocampal function was shown [13, 16, 17]. There is robust evidence that the left hippocampus is the main hub for verbal and episodic memory [34, 35]. However, there is still controversial and intensive discussion whether and to what extent hippocampal memory is material-specific or not. Regarding spatial navigation and memory, the data existing so far are contrastive. [14, 15, 17] Studies on patients with mesial temporal lobe epilepsy and status post anterior temporal lobe resection for refractory epilepsy suggest that spatial navigation and memory is particularly a function of the right hippocampus, thus being complementary to verbal and episodic memory as the primary function of the left hippocampus [13, 17, 34]. The right hippocampus seems to play a prominent role in allocentric spatial navigation and long-term storage of allocentric spatial memories [3, 4, 13]. Further support for this hypothesis derives from brain activation patterns when absolving a real-space navigation task with predominant activation of the right hippocampus in healthy subjects as compared to patients with amnestic mild cognitive impairment [19–21]. A quantitative meta-analysis on spatial navigation and episodic memory also deciphered a more prominent role of the right hippocampus for the retrieval of spatial memory as compared to episodic memory [3, 34]. Nevertheless, there are also various studies conflicting these findings. Particularly, one study on patients with mesial temporal lobe epilepsy due to hippocampal sclerosis or anterior temporal lobe resection challenges the view on material-specific lateralized hippocampal functions since there were no differences in performance between patients with sclerosis or resection of the left and right hippocampi during a VR-based navigation paradigm, object location task, and plan drawing [18].

The results of the current study partially reflect both of the above-described findings. On the one hand, quantitative measures such as total error rate for the whole real-space navigation paradigm, error rate for recombining novel routes, and retracing familiar routes highlighted the prominent role of the hippocampus for spatial navigation, particularly when novel routes have to be created. On the other hand, those quantitative measures did not differ between patients with right and left temporal lobe epilepsy after correcting for the identified confounders for spatial navigation performance (such as age, disease duration, and brain lesion status). Nevertheless, the quantitative performance in patients with rTLE was clearly worst among all the examined subgroups. In contrast to patients with lTLE and FLE, rTLE patients were the only subgroup with an inferior performance when retracing familiar routes. Furthermore, additional indicators of an optimized navigation strategy, such as duration at crossroads, unexplored territory, and use of shortcuts, further underpinned that side-specific hippocampal dysfunction in rTLE seems to affect navigational abilities in real space more profoundly than in lTLE. A comprehensive explanation of the complex behavioral findings in patients with TLE might come from a previous study, suggesting that both hippocampi provide complementary representations for spatial navigation rather than a single common function. The right hippocampus seems to be particularly important for encoding places, while the left hippocampus for temporal sequences, thus also building a bridge from spatial navigation and memory to episodic memory [3, 4].

One major advantage of our study is that all the epilepsy patients absolved a well-validated real-space navigation paradigm, for which engagement of a widespread multifaceted brain network was shown in previous studies with a particular role of the hippocampus [19–22]. However, our study also has limitations. First, the three epilepsy subgroups were not equal in age with rTLE patients being significantly older than lTLE and FLE patients. We therefore conducted an ANCOVA, thus correcting for age as a relevant confounder on navigation performance. An additional limitation is the clinical heterogeneity related to previous epilepsy surgery as our cohort included patients with previous surgical treatment of their epilepsy and not. However, those patients with epilepsy surgery were quite equally distributed within patient subgroups with rTLE, lTLE, and FLE in our cohort. Furthermore, our psychometric tests for executive functions and verbal memory were not designed to detect subtle deficits for these cognitive domains with potential impact on navigation performance. Therefore, we cannot exclude broader cognitive contributions to the observed navigation performances in our patients due to subtle specific deficits in certain important domains with relevance for spatial navigation, such as executive functions, detail-rich and complex visual memory, and other domains of higher-order visual perception and processing. Therefore, future studies should implement additional tests, such as the Brixton Spatial Anticipation Test or card-sorting paradigms, more complex test batteries for visual scene perception, and processing and test batteries for other domains of higher-order visual function such as VOSP battery. Lastly, it may be possible that some patients had subclinical epileptic activity, which may affect brain networks across lesion localization.

## Conclusion

In conclusion, patients with epilepsy (i.e., lTLE, rTLE, FLE) performed significantly inferior than HC in a standardized and well validates real-space navigation paradigm. This underpins that real-space navigation is indeed a multifaceted task comprising a complex brain network. TLE patients exhibited a significantly higher error rate, rTLE patients worse than lTLE patients. There were also relevant differences in the navigational path with longer stays at crossroads and inefficient unexplored territory as well as less application of a possible shortcut in the rTLE patients exclusively. These findings support the view that the hippocampus is a major hub for human navigation with a particularly prominent role of the right hippocampus for allocentric navigation. Our data also give rise that both hippocampi have functions for spatial navigation and that there seems no unequivocal lateralization of hippocampal function for different memory domains, such as spatial allocentric memory and episodic memory. Altogether, clinically well-characterized epilepsy patients seem to comprise a good collective to get deeper insights into human cognitive functions.

## Supplementary Information

Below is the link to the electronic supplementary material.

Supplementary file1 (DOCX 14 KB)

## Data Availability

The data that support the findings of this study are available upon reasonable request and in compliance with the local and international ethical guidelines.
