# Supplementary material for: Real-space navigation in patients with focal epilepsy underpins the prominent role of the hippocampus for allocentric navigation in humans
Source: J Neurol. 2026 Sep 27;273(10):626. doi: 10.1007/s00415-026-14164-7 (PMC13616842; doi:10.1007/s00415-026-14164-7)
Supplement: Supplementary file 1 [file 415_2026_14164_MOESM1_ESM.docx]

**Supplementary Table 1 Neuropsychological test performance**

| **Parameter** | **rTLE** | **lTLE** | **FLE** | **HC** | **ANOVA (F, p)** |
| --- | --- | --- | --- | --- | --- |
| 4 mountains test: score (n) | 9.3±2.4 | 9.7±2.7 | 9.0±3.7 | 9.9±3.8 | 0.2, 0.927 |
| 4 mountains test: time (s) | 140.9±42.1 | 128.1±34.3 | 170.9±37.5 | 117.0±30.5 | **3.5, 0.027*** |
| ROCF score: copy (n) | 35.2±1.3 | 35.6±0.7 | 35.7±0.5 | 35.8±0.4 | 0.7, 0.571 |
| ROCF score: after 3 min (n) | 22.0±5.0 | 24.4±6.8 | 24.8±5.4 | 23.8±6.9 | 0.3, 0.860 |
| ROCF score: after 30 min (n) | 22.8±4.7 | 25.3±6.5 | 23.5±6.0 | 25.2±5.9 | 0.3, 0.831 |
| CERAD word list 1 (n) | 5.6±1.6 | 7.3±2.6 | 7.4±1.1 | 7.8±1.8 | 2.4, 0.093 |
| CERAD word list 2 (n) | 8.0±1.4 | 8.4±2.1 | 9.3±1.0 | 8.3±2.0 | 0.9, 0.468 |
| CERAD word list 3 (n) | 9.0±1.5 | 8.8±2.1 | 9.4±0.9 | 9.0±1.5 | 0.2, 0.887 |
| CERAD word list 4 (n) | 7.1±2.6 | 7.4±3.4 | 9.0±1.1 | 8.5±2.3 | 1.0, 0.397 |
| TMT-B time (s) | 113.6±105.6 | 83.1±45.8 | 99.8±46.4 | 72.5±21.0 | 0.5, 0.663 |
| Semantic fluency (n) | 22.4±7.1 | 18.9±5.4 | 20.1±7.1 | 20.0±6.1 | 0.5, 0.694 |
